# Visual and somatosensory feedback mechanisms of precision manual motor control in autism spectrum disorder

**DOI:** 10.1186/s11689-021-09381-2

**Published:** 2021-09-08

**Authors:** Robin L. Shafer, Zheng Wang, James Bartolotti, Matthew W. Mosconi

**Affiliations:** 1 Life Span Institute, University of Kansas, Lawrence, KS USA; 2 Kansas Center for Autism Research and Training (K-CART), University of Kansas, Lawrence, KS USA; 3 Department of Occupational Therapy, University of Florida, Gainesville, FL USA; 4 Department of Applied Physiology and Kinesiology, University of Florida, Gainesville, FL USA; 5 Clinical Child Psychology Program, University of Kansas, Lawrence, KS USA

**Keywords:** Proprioception, Visual gain, Autism spectrum disorders, Sensorimotor, Sensory reweighting, Fine motor control, Entropy, Grip force

## Abstract

**Background:**

Individuals with autism spectrum disorder (ASD) show deficits processing sensory feedback to reactively adjust ongoing motor behaviors. Atypical reliance on visual and somatosensory feedback each have been reported during motor behaviors in ASD suggesting that impairments are not specific to one sensory domain but may instead reflect a deficit in multisensory processing, resulting in reliance on unimodal feedback. The present study tested this hypothesis by examining motor behavior across different visual and somatosensory feedback conditions during a visually guided precision grip force test.

**Methods:**

Participants with ASD (*N* = 43) and age-matched typically developing (TD) controls (*N* = 23), ages 10–20 years, completed a test of precision gripping. They pressed on force transducers with their index finger and thumb while receiving visual feedback on a computer screen in the form of a horizontal bar that moved upwards with increased force. They were instructed to press so that the bar reached the level of a static target bar and then to hold their grip force as steadily as possible. Visual feedback was manipulated by changing the gain of the force bar. Somatosensory feedback was manipulated by applying 80 Hz tendon vibration at the wrist to disrupt the somatosensory percept. Force variability (standard deviation) and irregularity (sample entropy) were examined using multilevel linear models.

**Results:**

While TD controls showed increased force variability with the tendon vibration on compared to off, individuals with ASD showed similar levels of force variability across tendon vibration conditions. Individuals with ASD showed stronger age-associated reductions in force variability relative to controls across conditions. The ASD group also showed greater age-associated increases in force irregularity relative to controls, especially at higher gain levels and when the tendon vibrator was turned on.

**Conclusions:**

Our findings that disrupting somatosensory feedback did not contribute to changes in force variability or regularity among individuals with ASD suggests a reduced ability to integrate somatosensory feedback information to guide ongoing precision manual motor behavior. We also document stronger age-associated gains in force control in ASD relative to TD suggesting delayed development of multisensory feedback control of motor behavior.

## Background

Autism spectrum disorder (ASD) is characterized by social-communication abnormalities and restricted, repetitive behaviors [1]. Additionally, deficits in sensorimotor behavior are highly prevalent in persons with ASD [2]. Sensorimotor deficits appear to emerge before the core social-communication and repetitive behavior symptoms of ASD [3, 4], and they are associated with the severity of social, communication, repetitive behavior, and cognitive symptoms [3, 5–8]. Sensorimotor deficits in ASD have been observed across a range of behaviors including gait [9, 10], postural control [11–13], precision gripping [14, 15], reaching [6, 16, 17], and eye movements [18–20]. They affect multiple stages of motor processing including motor planning [14], motor learning [6, 16, 17], and online motor control [14, 15]. Individuals with ASD show structural and functional brain differences in cerebellar-cortical sensorimotor networks [17, 21–24], which are associated with the severity of sensorimotor deficits [17, 23, 24]. Given the pervasiveness of sensorimotor issues in ASD, their early emergence, and their association with core symptoms, characterizing the nature and age-dependent differences in sensorimotor behaviors in ASD has great potential to provide new information on developmental processes that contribute to clinical outcomes. Additionally, distinct sensorimotor behaviors and control processes are subserved by discrete brain circuitries that are relatively ubiquitous across individuals and species, making them promising targets for identifying specific neurodevelopmental mechanisms associated with ASD.

Multiple studies have indicated that individuals with ASD show deficits in processing sensory feedback to reactively adjust ongoing motor behaviors. Across multiple effector systems, including upper [14] and lower limbs [11, 12], individuals with ASD show increased variability and regularity of continuous motor behaviors. Increased variability represents less consistency in the precision of motor output due to increases in intrinsic noise or reduced ability to reactively adjust output. Increased regularity of a motor output time series represents fewer degrees of freedom of the control system, or a reduced ability to integrate multiple control processes that operate on different time scales. Analyses of motor variability and regularity therefore provide unique information regarding the distinct motor control processes that may be disrupted in ASD.

Understanding sensorimotor control processes that are altered in ASD is important for clarifying mechanisms and determining more effective therapeutic approaches that may address multiple clinical and functional skill issues. This hypothesis is supported by findings that sensorimotor behavior is important for the development of adaptive skills [25, 26], as well as cognitive [27], social [28–30], and language development [31]. Deficits in sensorimotor control in ASD also are associated with poorer outcomes in cognition, daily living skills, and social and language ability. Fine motor behaviors in particular appear to be consistently affected in infants with ASD and associated with reduced visuospatial cognition, exploratory behavior, and social orienting [32]. Development of fine motor control likely is especially central to developmental abilities due to its involvement in multiple aspects of daily function, including the abilities to grasp and manipulate objects and explore the environment—critical skills for early language and social development. Consistent with this hypothesis, more severe manual motor impairments in children with ASD are predictive of worse language outcomes in early childhood [33, 34] and reduced daily living skills in adolescence and adulthood [35]. Despite these findings, the motor control processes that disrupt fine manual motor control in ASD have not yet been determined.

Several studies show that motor deficits in persons with ASD are associated with atypical sensory feedback processing during behavior. Feedback processing differences in ASD have been observed in multiple sensory modalities. In studies of motor learning, individuals with ASD learn to adapt to proprioceptive errors more efficiently than typically developing (TD) controls indicating that they are over-reliant on proprioceptive feedback for motor learning [6, 16, 17]. In our studies of visually guided fine motor control, participants with ASD showed elevated motor variability and regularity compared to TD controls during precision gripping, especially when visual feedback was enhanced (high visual gain) or degraded (low visual gain) [14], indicating that they are over-reliant on visual feedback even when it was degraded or amplified. While these findings may appear contradictory, they suggest that in ASD sensory feedback processing deficits during motor behavior may differ according to the behaviors that are targeted and their relative reliance on separate sensory modalities.

We hypothesize that behavior-specific findings of visual or somatosensory bias in ASD suggest that sensorimotor deficits are not specific to a sensory domain but may instead be task-dependent and reflect difficulties integrating information across sensory domains to dynamically adjust motor output. Consistent with this hypothesis, several studies have found that individuals with ASD show deficits in multisensory integration, even though processing of simple, unimodal stimuli is largely intact [36–39]. During postural control—for which proprioceptive feedback is primary—individuals with ASD show elevated variability of their center of pressure (COP) when proprioceptive feedback is perturbed (tendon vibration), whereas TD controls are able to compensate for disrupted proprioceptive feedback by relying more heavily on a secondary source of feedback (in this case, visual) to minimize COP variability [13]. These results indicate that individuals with ASD are unable to reweight different sources of sensory feedback (i.e., up-weight secondary sources) in response to perturbations of the primary sensory input; however, reweighting of visual and somatosensory feedback has not been systematically assessed during visually dominant fine motor behavior in individuals with ASD. This work is needed as the ability to use sensory feedback to adjust fine motor behavior is important for conducting activities of daily living such as feeding, personal hygiene, dressing, and tool use, which often require both visual and somatosensory inputs to perform skillfully.

The present study manipulated visual and somatosensory feedback within a visually guided precision gripping task to assess how each feedback source influenced motor control in individuals with ASD. The precision gripping test used here involves continuous visual feedback, which has been shown to be the primary sensory feedback source for online control of visually guided upper limb movements [40–42]. We expected individuals with ASD would show increased variability and regularity during precision gripping relative to controls, especially when visual (primary) feedback was enhanced or degraded. This finding would support the hypothesis that individuals with ASD have difficulty down-weighting feedback from the primary sensory domain for visually guided movement. We also expected that force variability and regularity in individuals with ASD would be minimally impacted when somatosensory feedback was manipulated with tendon vibration, consistent with a reduced ability to utilize secondary sources of sensory feedback to optimize motor output.

## Methods

### Participants

Forty-three participants with ASD (11 females) and 23 TD controls (12 females) matched on age (range 10–20 years) and handedness completed tests of precision gripping with their dominant hand (Table 1). Participants with ASD were recruited through our research registries comprised of individuals evaluated through the University of Kansas Health System who have consented to be contacted for research purposes, and though community advertisements. TD controls were recruited through community advertisements. ASD diagnoses were confirmed based on Diagnostic and Statistical Manual of Mental Disorders, Edition 5 (DSM-V) [1] criteria using the Autism Diagnostic Observation Schedule, Second Edition (ADOS-2) [43], Autism Diagnostic Interview – Revised (ADI-R) [44], and expert clinical opinion. Participants with ASD were excluded if they had a known genetic or metabolic disorder associated with ASD (e.g., fragile X syndrome) or a full-scale intelligence quotient (IQ) below 60 as measured using the Wechsler Abbreviated Scales of Intelligence, Second Edition (WASI-II) [45]. TD participants were excluded if they scored >8 on the Social Communication Questionnaire [46]; reported a history of psychiatric or neurologic disorders; had a family history of ASD in first-, second-, or third-degree relatives; had a family history of a developmental or learning disorder, psychosis, or obsessive compulsive disorder in first-degree relatives; or had a full-scale IQ below 85 as measured using the WASI-II. Participants also were excluded if they had a history of head injury, birth injury, or seizure disorder. No participants were taking medications known to affect sensorimotor behavior, including antipsychotics, stimulants, or anticonvulsants at the time of testing [47]. All participants had corrected or uncorrected visual acuity of at least 20/40. Adult participants provided written informed consent after a complete description of the study, in accordance with the Declaration of Helsinki and the approved University of Kansas Medical Center Institutional Review Board study protocol (IRB#: STUDY00140269). For participants under the age of 18 and adults who were under legal guardianship, a parent or legal guardian provided written informed consent on behalf of the participant, and the participant provided written assent. All study procedures were approved by the local Institutional Review Board.

**Table 1:** Demographic and clinical characteristics of individuals with ASD and TD controls

	**ASD**			**TD**			
	*N*	Ratio		*N*	Ratio		*χ*^2^
Sex	43	32M:11F	–	23	11M:12F	–	12.19*
Handedness	43	6L:37R	–	23	2L:21R	–	1.50
	*N*	Mean	SD	*N*	Mean	SD	t
Age	43	13.90	2.59	23	14.99	3.23	−1.39
ADOS-CSS	43	6.21	2.11	–	–	–	–
VIQ	40	96.15	19.06	23	107.70	10.33	−3.12*
PIQ	41	101.22	16.26	23	111.65	12.37	−2.88*
SP-2: visual	29	15.41	5.29	–	–	–	–
SP-2: movement	29	18.41	6.17	–	–	–	–
Adolescent/adult SP: visual	11	25.36	5.84	–	–	–	–
Adolescent/adult SP: movement	11	18.82	5.12	–	–	–	–
BOT-2: fine motor control	40	42.9	10.51	–	–	–	–
MVC	43	51.58	20.12	23	64.81	33.09	−1.75*

*ASD* autism spectrum disorder, *TD* typical development, *ADOS-CSS* Autism Diagnostic Observation Schedule Composite Severity Score, *VIQ* verbal IQ, *PIQ* Perceptual (non-verbal) IQ, *SP(-2)* Sensory Profile (Second Edition), *BOT-2* Bruininks-Osteresky Test of Motor Proficiency, Second Edition, *N* sample Size, *SD* standard deviation, *MVC* maximum voluntary contraction

*p < .05

Participants with ASD completed either the Sensory Profile, Second Edition [48] (SP-2; participants up to age 14 years) or the Adolescent/Adult Sensory Profile [49] (Adolescent/Adult SP; participants 14 years and older) and the Bruininks-Osteretsky Test of Motor Proficiency, Second Edition [50] (BOT-2) to assess clinical severity of sensory symptoms and motor deficits, respectively. Scores for the two versions of the SP are not standardized across versions, so summary statistics and analyses are separated according to test version. For the ADOS-2 and the SP, higher scores reflect more severe symptoms. On the BOT-2, higher scores reflect better performance.

### Precision grip testing

**Fig. 1:**
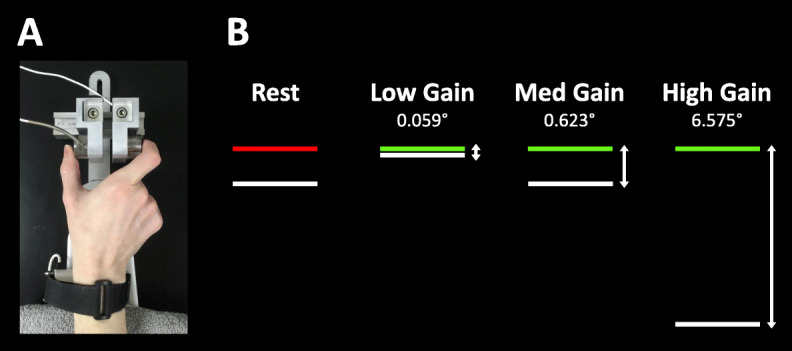
Task design. **a** Participants rest their arm on a custom arm rest with a tendon vibrator secured to their wrist with a Velcro strap. They place their thumb and index finger on the load cells of the force transducer. The tendon vibrator is either turned on to disrupt somatosensory feedback, or it is turned off so that there is no somatosensory disruption. **b** Participants view two bars on the computer screen. Participant force output is represented by the white bar, which moves up with increased force. The target bar is red during rest periods, and it turns green to indicate the start of the trial. Participants are instructed to press on the force transducers as quickly as possible when the target bar turns green and try to keep the white force bar at the same level as the green target bar. The gain of the visual feedback is presented at three different gain levels, such that the white force bar moves more per Newton of force at higher gain levels. At rest, the force output bar is at the 0N position, which changes as a function of the gain condition (shown here at medium gain)

Participants completed tests of precision gripping in a darkened room while seated 52cm from a 67cm (27in) Samsung LCD display monitor with a resolution of 1920×1080 and a 120-Hz refresh rate (Fig. 1). Participants sat with the elbow of their dominant hand comfortably positioned at 90 deg and their forearm resting in a custom arm brace fixed to the table to provide stability during testing. To assess precision grip behavior when somatosensory feedback was disrupted, participants completed grip testing with a tendon vibrator (VB 115, Techno Concept, Cereste, France) securely fastened on their wrist. A velcro strap held the tendon vibrator in place against the long finger flexor tendons. Towels were placed underneath the participants’ wrist to cushion the tendon vibrator from the surface of the table. The participants used their thumb and index finger of their dominant hand to press against two opposing precision load cells (ELFF-B4-100N; Entran) 1.27cm in diameter that were secured to a custom grip device attached to the arm brace. A Coulbourn (V72-25) resistive bridge strain amplifier received analog signals from the load cells. Data were sampled at 100 Hz with a 16-bit analog-to-digital converter (DI-720; DATAQ Instruments) and converted to Newtons of force using a calibration factor derived from known weights before the study [14].

Prior to precision grip testing, participants completed an assessment of their maximum grip strength, or maximum voluntary contraction (MVC) using their dominant hand. Participants completed three trials in which they were asked to press as hard as they could for three seconds. The average of the participant’s maximum force output across these trials comprised their MVC. During precision grip testing, the target force is set to a fixed percentage of the participants’ MVC to control for differences in strength across participants and ensure similar levels of relative exertion or fatigue across participants.

During the precision gripping task, participants viewed two horizontal bars on the screen (Fig. 1B). A horizontal white force bar moved upward with increased force and downward with decreased force, and a static bar representing the target force was red during periods of rest and turned green to cue the participant to begin pressing at the beginning of each trial. Participants were instructed to press the load cells as quickly as possible when the red target bar turned green and to keep pressing so that the white force bar stayed as steady as possible at the level of the green target bar.

To test the impact of different sensory feedback processes on grip force behavior, participants completed testing across multiple levels of visual and somatosensory feedback. As in our previous study [14], visual feedback was manipulated by changing the visual gain of the white force bar (i.e., the vertical distance measured in visual angle that the force bar moved in response to a unit of change in force output). For example, for the three visual gain conditions used in the present study, the force bar moved upward 0.059° per 1N increase in force output at the lowest visual gain, 0.623° per 1N increase in force at medium visual gain, and 6.575° per 1N increase in force at the highest visual gain. These gain levels were selected based on findings from Vaillancourt et al. [51] that showed increases in force variability and regularity as visual angle increased up to 1°, beyond which force variability and regularity were relatively constant.

Somatosensory feedback was manipulated by applying tendon vibration to the underside of the wrist (long finger flexors) during gripping. The tendon vibrator at frequencies of at least 40 Hz alters the somatosensory percept by artificially stimulating mechanoreceptors in muscle spindle Ia afferents, which monitor muscle stretch [52]. At short durations (<25s), vibration increases the firing rate of Ia afferents, eliciting a proprioceptive illusion of muscle stretch in the agonist muscles [52–54], which has been demonstrated in the finger extensors [55]. At prolonged durations (>25s), vibration fatigues the Ia afferents resulting in reduced firing rates [53, 54]. In both situations, the vibration disrupts the natural stimulation of the Ia afferents, resulting in inaccurate perception of the posture of the stimulated effector [53]. Muscle spindle afferents also monitor applied load (force against the limb) [56]. Unlike in the elbow joint, where applied load and muscle stretch interact to produce a proprioceptive percept of limb position, these inputs are processed independently in the fingers [56]. Therefore, vibration of the finger flexors may affect perception of finger posture, force against the fingers, or both, so the effects may not be specific to proprioception as it is in studies of other joints. For this reason, we refer to the use of tendon vibration as a manipulation of somatosensory feedback rather than proprioceptive feedback. Participants completed precision grip trials with the tendon vibrator turned on at a frequency of 80 Hz based on prior research suggesting multiple motor behaviors are reliably disrupted at 80 Hz [57]. The 80 Hz vibration applied to the forearm side of the wrist in our experimental paradigm targets the long finger flexors to disrupt the somatosensory perception of the index finger. Participants also completed trials while wearing the tendon vibrator turned off (no disruption to somatosensory feedback) keeping wrist position consistent across conditions.

Participants completed blocks of 5 trials at each gain level and tendon vibration frequency using their dominant hand (5 trials × 3 gain levels × 2 vibration conditions = 30 trials). Trials were 15s in duration and alternated with 15-s rest periods. Each block was separated by 30s of rest. The target force was set to 45% of the participant’s MVC for all trials. The tendon vibration off condition was always administered prior to the on condition as vibration effects on motor control can persist for at least 20 min after the tendon vibration is turned off [58]. The order of gain levels was randomized across participants.

### Data processing

Force traces for each trial were low-pass filtered via a double-pass fourth-order Butterworth filter at a low-pass cutoff of 15 Hz in MATLAB (MathWorks, Inc., Natick, MA). Data were processed using a custom MATLAB scoring program previously developed by our lab [15]. To account for variability in the rate at which participants reached the target force, a minimum of 8s and a maximum of 12s of the 15-s trial data (from start cue to stop cue) were used for analyses. Trials were excluded if they had less than 8 seconds of sustained force output, the load cells were not properly re-zeroed between trials, or if there were indications that the participant was not following instructions (e.g., the mean force exceeded twice the target force, the mean force was less than half of the target force, there was evidence that the participants used fingers other than their index finger and thumb to press). Based on these criteria, 10.0% of trials were excluded. Force data were linearly detrended to account for drift in participants’ force output over the duration of the trial. The mean force of the trial divided by the target force was used as a measure of force accuracy. To assess force variability, the standard deviation (SD) of the force time series was examined. To test the time-dependent regularity of the force time series, sample entropy (SampEn) was calculated for each trial [59, 60]. SampEn is defined as the natural logarithm of the conditional probability that two similar sequences of *m* data points in a timeseries of a given length (*N*) remain similar within a tolerance level (*r*) at the next data point in the series. SampEn returns a value between 0 and 2. Lower values of SampEn indicate greater regularity of the timeseries (e.g., a sine wave, with its predictable oscillating pattern, would have a SampEn value near 0). SampEn has been shown to be stable with as few as 200 data points in the timeseries. Parameter settings for SampEn calculations were *m* = 2 and *r* = .2 × SD of the timeseries. The timeseries length ranged from 800 to 1200 data points (8–12 s sampled at 100 Hz). The sampenc.m function (for MATLAB) from the PhysioNet Toolbox was used [61, 62] to calculate SampEn values for each trial.

### Statistical analysis

Force accuracy, SD, and SampEn were analyzed using separate linear multilevel mixed effects models (MLM) [63, 64]. MLM allows for the analysis of within- and between-subjects fixed effects while allowing within-subjects effects to vary randomly and is robust to missing data. Gain level (low, medium, high) and vibration condition (on, off) were included as level 1 predictors. Group (ASD, TD), age, sex, and perceptual IQ (PIQ) were included as level 2 predictors. Random intercepts of participant also were included in our models.

Initial models included three-way interactions of Group × Gain Level × Vibration Condition, Group × Gain Level × Age, and Group × Vibration Frequency × Age, as well as all relevant 2-way interactions and main effects terms. To maintain the most parsimonious models possible, other 3-way and 4-way interactions were not included. Sex and PIQ effects also were tested in the models, as these variables significantly differed between groups. Models were fitted using the maximum likelihood approach to allow for model comparisons. Terms were removed systematically, and model fit was compared between the previous model and the model with the removed term using likelihood ratio tests. Terms that did not significantly improve model fit (*p* < 0.05), based on the model comparisons, were not included in the final models. Satterthwaite’s method was used to calculate degrees of freedom for the final model and post hoc comparisons [65]. Due to the inherent challenge in determining denominator degrees-of-freedom and calculating *p* values for MLMs, we treated the *t* value as a *z* value and used a *z* > 1.96 threshold as an additional guideline for determining whether terms explained significant variance in the model [65].

Simple coding was used for group (TD = − 0.5, ASD = 0.5), vibration condition (off = − 0.5, on = 0.5), and sex (male = − 0.5, female = 0.5). Simple coding was used for gain level with one coding system used to represent low gain (0.67) vs. medium and high gain comparisons (− 0.33), and another system used to represent high gain (0.67) vs. low and medium gain comparisons (− 0.33). Age was z-transformed, and SD was log transformed to correct for a skewed distribution. Based on this coding system, the intercept for each model represented the grand mean of the sample. Mixed effects modeling was conducted using the lme4 package within *R* version 4.0.0 [63].

Pearson correlations were used to assess the relation between experimental variables and ASD symptom severity measured using the ADOS Composite Severity Score (ADOS-CSS). To assess associations between precision force outcomes and sensory issues, the Visual Processing and Movement Processing subscales of the SP-2 and Adolescent/Adult SP were examined. Analyses for SP-2 (*N* = 29) and Adolescent/Adult SP (*N* = 11; three participants did not complete the Adolescent/Adult SP) were conducted independently as scores are not standardized across the two versions of this measure. Force variability and regularity also were examined in relation to the Fine Motor Control Subscale of the BOT-2. Three participants with ASD did not complete the BOT-2 (*N* = 40). *P* values were adjusted using false discovery rate (FDR) to limit Type I error for each set of correlations; however, due to small sample sizes and the exploratory nature of these analyses, interpretation of results focuses on effect sizes (*r* values).

## Results

### Force accuracy

Force accuracy did not differ between groups; however, females were more accurate than males (*β* = 0.045, *R*^2^ = .048, *t*_63.7_ = 2.29, *p* = .0253). Participants were more accurate during medium and high visual gains compared to low gain (*β* = − 0.0439, *R*^2^ = .036, *t*_301.2_ = − 5.061, *p* <.0001), and accuracy improved with age (*β* = 0.021, *R*^2^ = .046, *t*_63.4_=2.23, *p* = .0293).

### Force variability

**Fig. 2:**
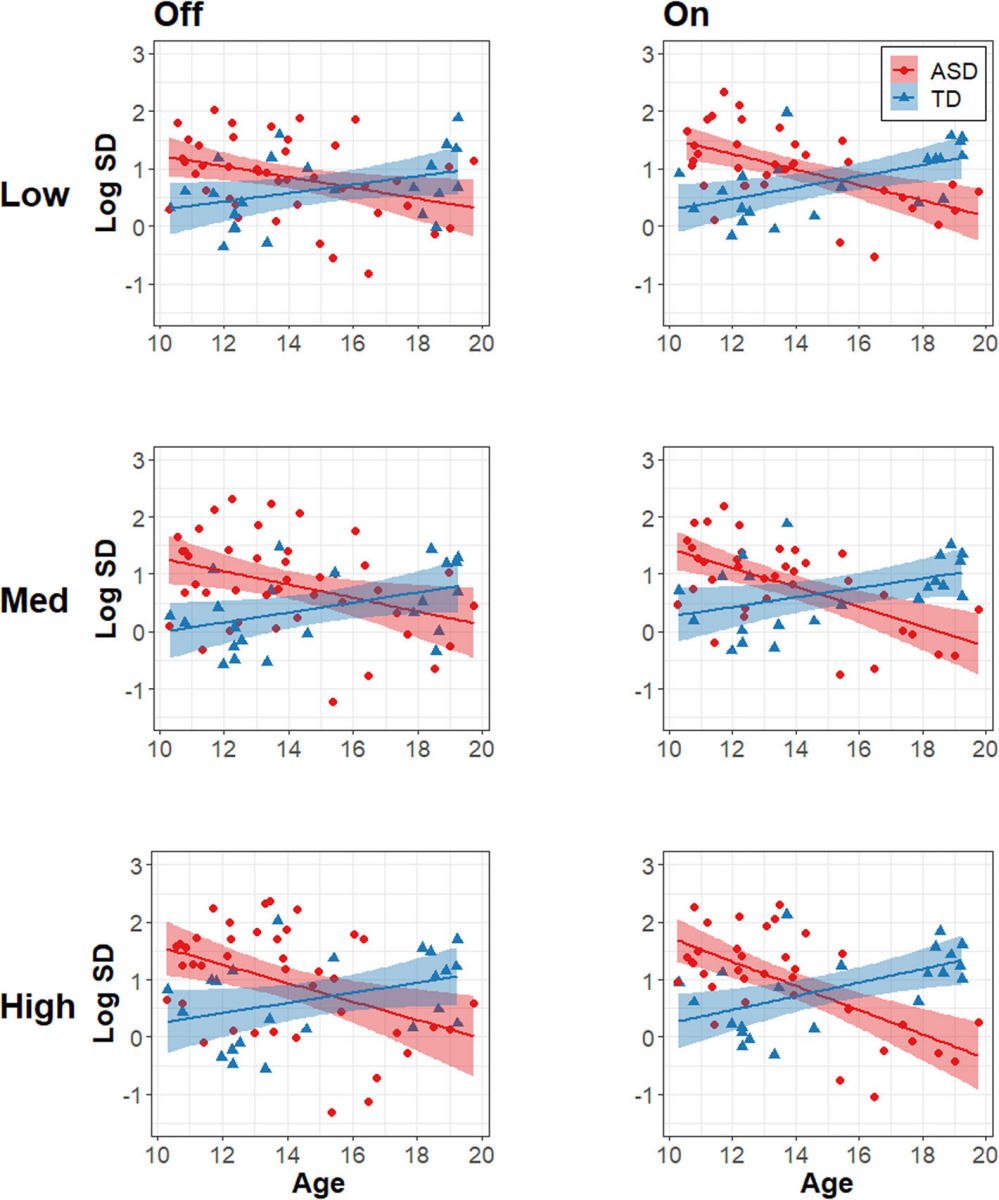
Force variability vs. Age. Age associations with the log of force SD for the ASD (red circles) and TD (blue triangles) groups. Columns represent tendon vibration off (left) and on (right). Rows represent low (top), medium (middle), and high (bottom) gain levels. Age is in years. Shaded areas represent the 95% confidence intervals

**Fig. 3:**
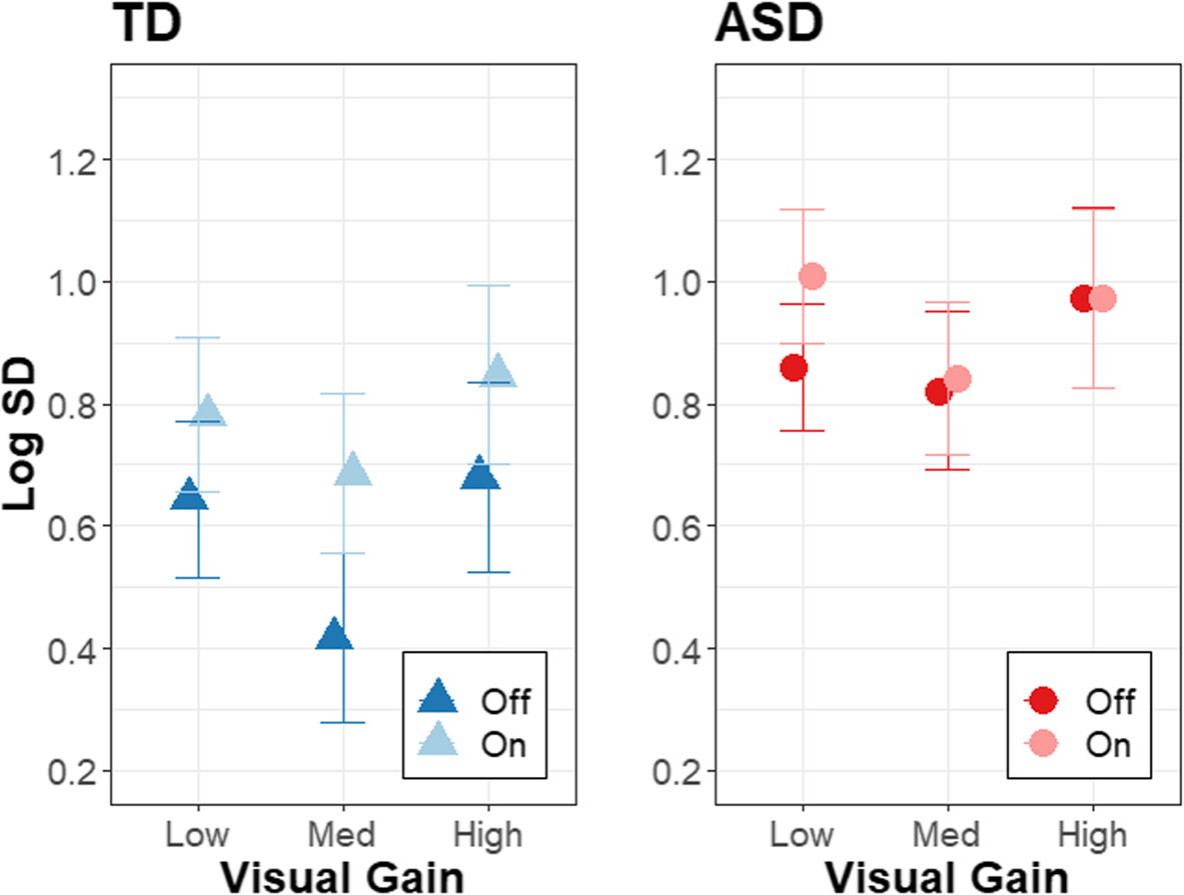
Condition effects on force variability. Effects of tendon vibration (off: dark, on: light) and gain level on the log of force SD for the ASD (red circles) and TD (blue triangles) groups. Error bars represent standard error

The results of the model for force SD are summarized in Table 2. Group differences in force SD varied as a function of age (Fig. 2; *β* = − 0.573, *R*^2^ = .168, *t*_65.6_ = − 4.054, *p* = .0001) and tendon vibrator condition (Fig. 3; *β* = − 0.157, *R*^2^ = .004, *t*_304.6.0_ = − 2.062, *p*= .0400). Follow-up comparisons of marginal slopes indicated that force SD decreased with age in the ASD group but not in the TD group (*β*_ASD_ = −0.310, *β*_TD_ = 0.263). Comparison of estimated marginal means indicated that TD controls showed higher force SD with the tendon vibrator on compared to off (*t*_303_ = −3.372, *p* = .0008), whereas individuals with ASD showed similar levels of force SD with the tendon vibrator turned on and off (*t*_307_ = −0.960, *p* = .3376).

**Table 2:** Linear mixed effects model summary for force standard deviation (variability)

	**Fixed effects**	**Estimate (SE)**	**df**	***t***	**Partial*R***^**2**^
**Log SD**	*Intercept*	.698 (.0744)	65.4	9.383***	
	*Level 1*				
	Gain_Low vs. Med & High_	.114 (.0439)	301.9	2.595**	.006
	Gain_Low & Med vs. High_	.169 (.0443)	301.9	3.826***	.012
	Vibration	.123 (.0380)	304.5	3.247**	.009
	*Level 2*				
	Group	.145 (.1498)	65.6	.966	.011
	Age	− .023 (.0715)	65.5	−.328	.001
	Sex	−.398 (.1532)	65.8	−2.599*	.076
	*Interactions*				
	Group × Vibration	−.157 (.0760)	304.6	−2.062*	.004
	Group × Age	−.573 (.1413)	65.6	−4.054***	.168
	**Random effects**	**Variance (SD)**			
	*Participant (intercept)*	.282 (.5313)			
	Residual	.119 (.3443)			

*SD* standard deviation, *SE* standard error

*p < .05, **p < .01, ***p < .001

### Force regularity

**Fig. 4:**
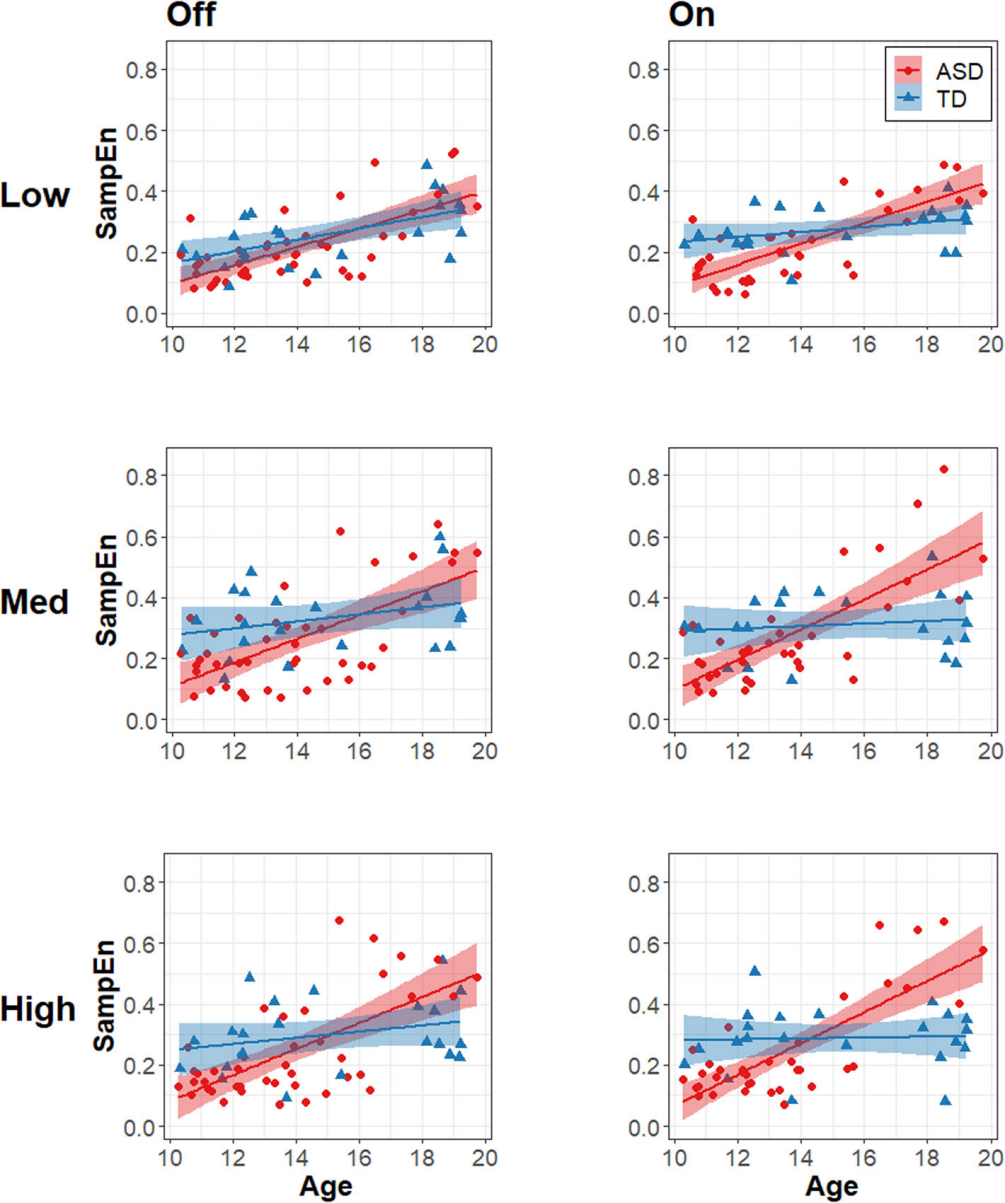
Force regularity vs. age. Age associations with the force SampEn for the ASD (red circles) and TD (blue triangles) groups. Columns represent tendon vibration off (left) and on (right). Rows represent low (top), medium (middle), and high (bottom) gain levels. Age is in years. Shaded areas represent the 95% confidence intervals

**Fig. 5:**
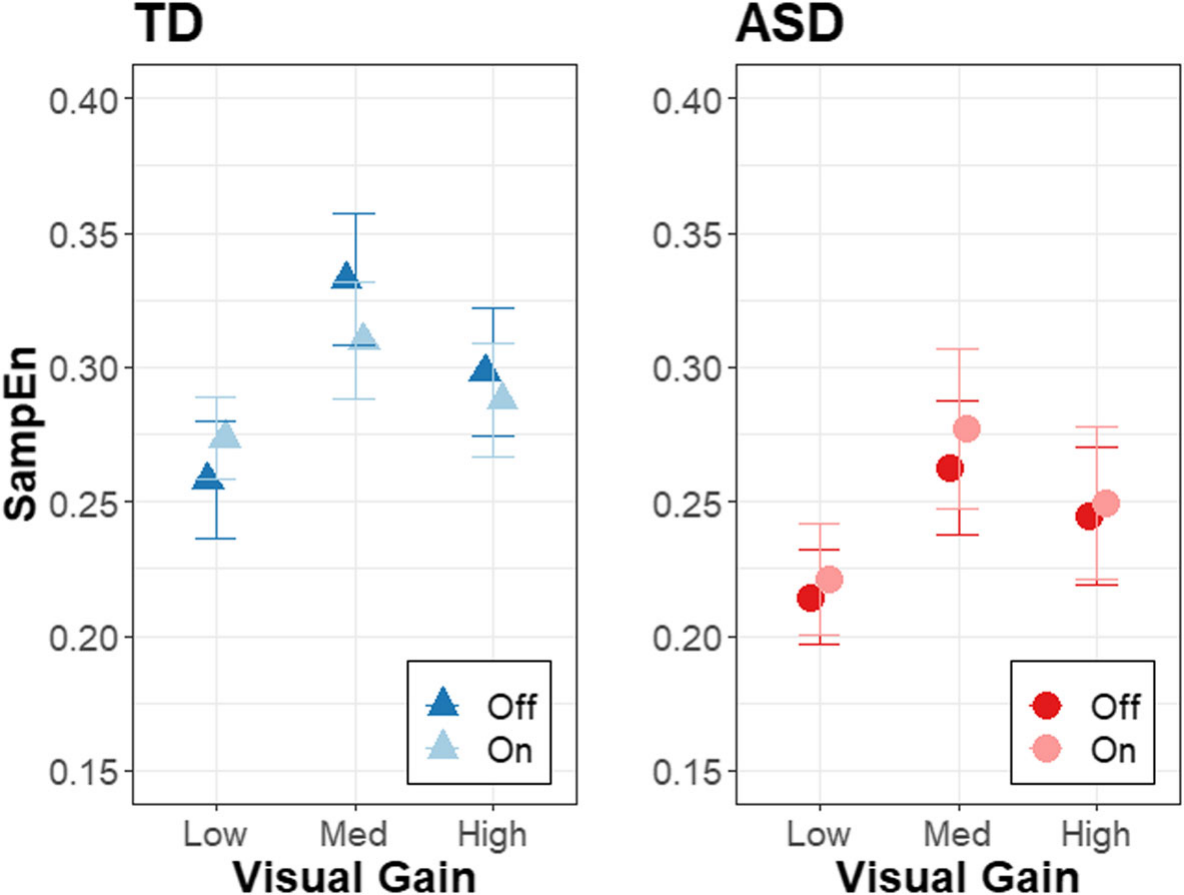
Condition effects on force regularity. Effects of tendon vibration (off: dark, on: light) and gain level on the force SampEn for the ASD (red circles) and TD (blue triangles) groups. Error bars represent standard error

Force regularity varied as a function of age, but the strength of this relationship differed between groups and was dependent on visual gain level (Table 3; *β*_Group × Gain Low vs. Med & High x Age_ = −0.0540, *R*^2^ = .011, *t*_301.3_ = −3.223, *p* = .0014) and tendon vibration condition (*β*_Group x Vibration x Age_ = 0.0342, *R*^2^ = .007, *t*_303.4_ = 2.460, *p* = .0144). Follow-up comparisons of marginal slopes indicated that individuals with ASD showed stronger age-associated increases in SampEn than TD individuals at medium (Fig. 4; *β*_ASD_ = 0.124, *β*_TD_ = 0.0237, *t*_91.7_ = 4.189, *p* = .0009) and high gain levels (*β*_ASD_ = 0.135, *β*_TD_ = 0.0183, *t*_93.1_ = 4.841, *p* = .0001), but not at low gain. Group × Age × Vibration interaction effects reflected stronger age-related increases in SampEn for individuals with ASD relative to TD individuals with the tendon vibrator off (*β*_ASD_ = 0.109, *β*_TD_ = 0.0383, *t*_76.3_ = 3.090, *p* = .0028) that were even more pronounced with the tendon vibrator on (*β*_ASD_ = 0.121, *β*_TD_ = 0.0167, *t*_79.5_ = 4.537, *p* < .0001) as TD individuals did not show increases in SampEn with age. Group and condition effects are depicted in Fig. 5.

**Table 3:** Linear mixed effects model summary for force sample entropy (irregularity)

	**Fixed effects**	**Estimate (SE)**	**df**	***t***	**Partial*R***^**2**^
**SampEn**	*Intercept*	.274 (.0114)	64.7	24.110***	
	*Level 1*				
	Gain_Low vs. Med & High_	−.0598 (.0088)	301.0	−6.835***	.050
	Gain_Low & Med vs. High_	−.0209 (.0088)	301.0	−2.376*	.006
	Vibration	.0053 (.0073)	305.9	.720	.001
	*Level 2*				
	Group	−.0209 (.0227)	64.7	−.921	.009
	Age	.0713 (.0109)	64.4	6.521***	.324
	*Interactions*				
	Group × Gain_Low vs. Med & High_	.0012 (.0175)	301.0	.068	<.001
	Group × Gain_Low & Med vs. High_	.0120 (.0176)	301.0	.681	.001
	Group × Vibration	.0208 (.0147)	305.9	1.416	.002
	Group × Age	.0875 (.0219)	64.4	4.005***	.153
	Gain_Low vs. Med & High_ × Age	−.0102 (.0084)	301.3	−1.219	.002
	Gain_Low & Med vs. High_ × Age	.0027 (.0085)	301.3	.318	<.001
	Vibration frequency × Age	−.0046 (.0069)	303.4	−.658	<.001
	Group × Gain_Low vs. Med & High_ × Age	−.0534 (.0168)	301.3	−3.223**	.011
	Group × Gain_Low & Med vs. High_ × Age	.0161 (.0169)	301.3	.955	.001
	Group × Vibration × Age	.0342 (.0139)	303.4	2.460*	.007
	**Random effects**	**Estimate (SD)**			
	*Participant (intercept)*	.0067 (.0816)			
	Residual	.0042 (.0645)			

*SD* standard deviation, *SE* standard error

*p < .05, **p < .01, ***p < .001

### Correlations with symptom severity

Correlations between force SD and clinical ratings are shown in Table 4. The Movement Processing subscale of the SP-2 was positively trending with force SD in the tendon vibrator off condition (*r* = .38, *p* = .09) and the low (*r* = .41, *p* = .07) and medium visual gain conditions (*r* = .39, *p* = .06). Force SD was not correlated with the SP-2 Movement Processing subscale for any other conditions, and SD did not correlate with the SP-2 Visual Processing subscale for any visual gain or tendon vibration conditions. The BOT-2 Fine Motor Control Subscale showed negative trends with force SD in the tendon vibrator on (*r* = −.41, *p* = .06) and medium visual gain (*r* = −.38, *p* = .06) conditions. Force SD correlations with the ADOS-CSS and the Movement and Visual Processing subscales of the Adolescent/Adult SP did not survive FDR corrections, though effect sizes indicated moderate associations (*r*>0.3) for some sensory conditions, including tendon vibration off, and all visual gain conditions (Table 4). Force SampEn correlations did not survive FDR corrections for any clinical measures or sensory conditions, though effect sizes, reported in Table 5 indicted moderate correlations for some conditions including tendon vibration on and medium visual gain.

**Table 4:** Associations between force variability and clinical symptoms across visual gain and somatosensory feedback conditions

Force variability (Log SD)	Off		On		Low gain		Med gain		High gain	
	*N*	*r*	*N*	*r*	*N*	*r*	*N*	*r*	*N*	*r*
ADOS-CSS	43	.337	37	.188	43	.334	43	.331	42	.220
SP-2: visual	29	.094	26	.108	29	.240	29	.164	29	.060
Adolescent/Adult SP: visual	11	.053	9	.207	11	−.030	11	−.008	11	.076
SP-2: movement	29	.346	26	.145	29	.379	29	.353	29	.191
Adolescent/Adult SP: movement	11	.313	9	−.122	11	.180	11	.124	11	.406
BOT-2: fine motor control	40	−.306	35	−.406	40	−.309	40	−.376	39	−.293

*SD* standard deviation, *N* sample size, *R* Pearson correlation coefficient, *ADOS-CSS* Autism Diagnostic Observation Schedule Composite Severity Score, *SP* Sensory Profile, *BOT-2* Bruininks-Osteretsky Test of Motor Proficiency, Second Edition

**Table 5:** Associations between force irregularity and clinical symptoms across visual gain and somatosensory feedback conditions

Force irregularity (SampEn)	Off		On		Low gain		Med gain		High gain	
	*N*	*r*	*N*	*r*	*N*	*r*	*N*	*r*	*N*	*r*
ADOS-CSS	43	−.111	37	.016	43	−.083	43	−.089	42	−.080
SP-2: visual	29	.004	26	.046	29	−.061	29	−.108	29	.073
Adolescent/Adult SP: visual	11	.085	9	.311	11	.217	11	.296	11	.163
SP-2: movement	29	−.128	26	.309	29	−.040	29	−.100	29	.030
Adolescent/Adult SP: movement	11	−.100	9	.642	11	.117	11	.305	11	.020
BOT-2: fine motor control	40	.205	35	.259	40	.210	40	.248	39	.282

*SampEn* Sample Entropy, *N* Sample Size, *R* Pearson Correlation Coefficient, *ADOS-CSS* Autism Diagnostic Observation Schedule Composite Severity Score, *SP* Sensory Profile, *BOT-2* Bruininks-Osteretsky Test of Motor Proficiency, Second Edition

## Discussion

This was the first known study to systematically assess the distinct contributions of visual and somatosensory feedback on precision manual motor control in persons with ASD. Two key findings were identified. First, we found that disrupting somatosensory feedback (applying tendon vibration) during visually guided gripping led to significant increases in force variability for TD individuals only, suggesting individuals with ASD show reduced involvement of somatosensory (secondary) feedback to guide precision manual motor control. Second, force variability decreased with age in individuals with ASD only, indicating delayed maturation of visual feedback mechanisms of precision manual control. Similarly, age-associated increases in force irregularity (SampEn) were stronger in individuals with ASD than TD controls suggesting protracted development of motor control processes involved in integrating multisensory inputs that operate on different time scales.

### Sensory feedback processing during motor behavior in ASD

Our findings that only TD controls showed changes in force control during somatosensory feedback interference suggest that TD controls integrate feedback across visual and somatosensory modalities, but this multimodal integration is deficient in ASD. Multisensory feedback integration during motor behavior involves modulating the weighting of feedback from separate sensory modalities to optimize motor output [66]. Vision is dominant for visually guided upper limb and precision motor behaviors [40–42], though secondary sources also contribute to the refinement of behavioral output [67, 68], consistent with our finding that TD controls showed increased force variability when somatosensory feedback was perturbed. Individuals with ASD and TD controls showed similar changes in force variability when visual feedback was manipulated demonstrating that both groups used the primary feedback source during precision gripping. This finding also demonstrates that persons with ASD are able to modulate fine motor behavior using sensory feedback, but they predominantly rely on visual feedback rather than somatosensory feedback or a combination of both. Our previous studies of a similar precision gripping test indicated that individuals with ASD show more severe deteriorations in their ability to limit variability of force output when visual feedback is altered, further supporting the hypothesis that they are highly reliant on visual input (i.e., the dominant source of sensory feedback) for precision gripping [14, 69]. In the present study, individuals with ASD did not show elevations in force variability relative to TD controls that varied as a function of visual gain, perhaps reflecting the narrower range of visual gains and ages studied here relative to our prior work [14].

Our findings of decreased reliance on non-primary sensory feedback processes in ASD are consistent with prior studies of separate sensorimotor behaviors. For example, a study of postural control in ASD documented an over-reliance on proprioceptive feedback, which is the dominant sensory input for maintaining postural stability [70]. Specifically, Morris et al. [13] showed that disrupting proprioceptive feedback resulted in increased center of pressure (COP) variability in individuals with ASD regardless of whether visual feedback was available; however, TD controls only showed increased COP variability when both visual and proprioceptive feedback were disrupted. These results suggest that TD controls were able to compensate for disrupted proprioceptive feedback by up-weighting secondary sources of feedback (e.g., visual), whereas individuals with ASD continued to rely on the primary source of feedback (proprioceptive) even though it was unreliable. Combined with our findings, these results indicate that, individually, visual and somatosensory feedback mechanisms are relatively intact in ASD, but the ability to integrate and optimally weight feedback across multiple sensory modalities during motor behavior is compromised.

Motor learning studies also have demonstrated that persons with ASD are better at adapting to induced proprioceptive errors than TD controls during upper limb reaching, but they were less effective at adapting to visually induced errors [6, 16, 17]. On the surface, these studies seemingly contradict our finding that participants with ASD were under-reliant on somatosensory feedback. However, the prior motor learning studies assessed adaptation (changes to the motor plan) in response to external sensory perturbations, which is a fundamentally different behavioral process than monitoring and adjusting ongoing behavior during precision grip force and likely requires a different weighting of sensory feedback inputs. These studies provide evidence that deficits across diverse sensorimotor behaviors in persons with ASD reflect atypical weighting of sensory inputs and a reduced ability to integrate multiple sources of feedback.

### Development of sensorimotor control in ASD

We found that individuals with ASD show stronger age-associated gains in precision force control (decreased variability, increased entropy) relative to TD peers across all visual gain and tendon vibrator conditions. These results indicate that the development of precision sensorimotor control is delayed in ASD and that sensorimotor deficits (increased SD, reduced entropy) may represent important markers of neurodevelopmental dysfunction in childhood. Our findings are consistent with considerable evidence from infant sibling and early childhood studies that show sensorimotor deficits are some of the earliest indicators of ASD [71, 72] and may be most severe during the first years of life. While our data suggest sensorimotor impairments may be attenuated or even normalize by adolescence/early adulthood in ASD, their disruption early in life likely interferes with the maturation of cognitive, social, and language processes that are known to rely on early ontological progression of reaching and grasping behaviors [73–76]. Tracking the early childhood development of precision manual variability and regularity will be an important next step in characterizing key behavioral indicators of ASD and in defining neurodevelopmental mechanisms contributing to the range of clinical issues associated with ASD.

We also found that differences between individuals with ASD and TD peers in age-associated gains in force control varied across sensory feedback conditions suggesting distinct timing of separate sensory feedback control mechanisms. More specifically, age-related gains in motor variability (decreases) and irregularity (increases) were stronger in the ASD group during conditions in which visual feedback was most precise (higher gains). These findings are consistent with prior studies of normative development showing that while motor variability decreases and entropy increases with age, the rates and timing of these changes are dependent on the quality and nature of sensory feedback [77–79]. For example, no age-associated differences are seen in precision grip force variability and entropy across childhood and into adulthood (ages 6–22 years) when visual feedback is occluded, suggesting the ability to dynamically and precisely adjust motor behavior in response to sensory feedback improves with age due, at least in part, to a greater capacity to integrate multiple sensory inputs [77–79]. The stronger age-related improvements in force control that we observed in the ASD group relative to the control group suggest delayed maturation of sensory feedback processing for refining motor output. Unlike controls, age-related decreases in force regularity in the ASD group were similar across somatosensory feedback conditions indicating age-related improvements in the ASD group were dependent on the ability to utilize the dominant (visual) source of sensory feedback rather than the integration of multiple sensory modalities.

The age-associations observed in the present study differ from our prior precision gripping study, which found that TD individuals show greater improvements in motor regularity with age than individuals with ASD [14]. These opposing trends may be due to the age distributions in the samples. The prior study (range: 5–35 years, median: 13 years) likely captured a period of rapid maturation in TD children that also may represent an epoch of relatively slowed sensorimotor development in ASD. The present study restricted the age distribution to later childhood and early adulthood (range: 10–20 years, median 13.6 years) during a period in which typical motor development is relatively stable. The present findings, in addition to studies showing that motor deficits in ASD are more severe in early childhood and improve over the course of adolescence [80, 81], indicate that individuals with ASD experience a delayed trajectory of motor development.

### Implications for understanding neurodevelopmental processes associated with ASD

Our findings of sensorimotor impairment in ASD and reduced integration of multisensory feedback implicate dysfunction of cortical-cerebellar sensorimotor networks. Posterior parietal cortex, including superior and inferior parietal lobules, integrate multiple sensory inputs during motor behavior [82–84] and innervate premotor and primary motor cortices to generate reactive motor adjustments based on feedback error information [85–87]. Parietal-cerebellar circuits also form a faster subcortical pathway for translating sensory error information into corrective motor commands relayed to motor cortex [88, 89]. During motor behavior, cerebellar circuits critically compare the expected sensory consequences of motor output (received from primary motor cortex) to the actual consequences of the behavior (processed initially by primary and association sensory cortex) to correct errors in the motor command, which are relayed to the primary motor cortex though the thalamus [90, 91]. Our findings that persons with ASD relied almost exclusively on visual feedback during precision motor control suggest deficits in parietal-cerebellar networks that are responsible for integrating feedback from multiple sources to accurately update motor commands. Additionally, stronger age-related improvements in force regularity at higher visual gains in the ASD group suggest delayed development of cortical-cerebellar circuits involved in rapid visual feedback and feedback error processing.

Our prior fMRI studies have found increased activation of putamen and cerebellum in ASD relative to TD controls during precision gripping behavior, indicating greater reliance on subcortical sensorimotor processes [23]. Unlike controls, individuals with ASD showed no association between force variability and premotor activation, indicating that they do not modulate cortical motor planning circuits in response to sensory feedback [23]. At rest, individuals with ASD show increased functional connectivity (FC) relative to TD controls in cerebellar-occipital and cerebellar-parietal networks involved in visual and sensorimotor processing and reduced FC in cerebellar-frontal and cerebellar-temporal networks involved in cognitive and multisensory processing [24]. An independent study similarly found increased intrinsic FC between cerebellum and sensorimotor regions of cortex and reduced FC between cerebellum and cognitive regions of cortex, implying that persons with ASD rely on basic sensory processing rather than complex multisensory or executive processing for sensorimotor control [92]. However, others have found evidence of reduced intrinsic FC between sensorimotor cerebellum and parietal cortex, in persons with ASD, which was associated with praxis deficits [93] and the severity of clinical symptoms [94]. These findings implicate reorganization of cortical and subcortical sensorimotor networks in persons with ASD potentially resulting from delayed maturation and specialization, but studies of functional connectivity during sensorimotor behavior are necessary for determining the neural processes underlying motor issues in ASD.

### Sensorimotor behavior and clinical impairments

We found that force variability and regularity explained 9 to 15% of variability in clinically rated ASD symptom severity suggesting that sensorimotor feedback deficits may contribute to core symptoms or share common developmental pathways. For example, learning and interpreting social gestures requires early advances in sensorimotor behavior that facilitate both action representations, imitation, and reciprocal social interactions. More specifically, early developing sensorimotor processes involve integration of visual information regarding the timing and intention of others’ movement and mapping this information onto internal sensorimotor representations to estimate the expected visual and somatosensory consequences of the movement [28, 29]. Difficulties integrating visual and somatosensory feedback for motor control in ASD may not only impact self-generated movements, including socially relevant behaviors, but also compromise the developing child’s ability to interpret and predict others’ behaviors [30]. Further, our findings that more severe force control impairments in ASD are associated with clinical measures of motor ability indicate deficits of multisensory feedback control may contribute to functional motor issues in ASD.

### Limitations and future directions

Several limitations of the present study should be noted. First, the inclusion of younger children in future studies will be important for characterizing key epochs of sensorimotor dysmaturation in ASD. Second, while Morris et al. [13] demonstrated that persons with ASD were susceptible to the vibration induced proprioceptive illusion during a postural control task, and the vibration induced illusion has been elicited in the fingers of typically developing adults [55], the use of tendon vibration to disrupt proprioceptive/somatosensory feedback has not been demonstrated during fine motor behavior in persons with ASD. Frequency thresholds for disrupting the somatosensory percept may vary across individuals and groups; therefore, a focus of future research is to assess frequency thresholds for the somatosensory disruption within participants and use these individualized thresholds to apply tendon vibration at supra- and sub-threshold frequencies during precision gripping. Our study also did not include a sham vibration condition (i.e., vibration on at a frequency that does not disrupt somatosensory feedback), so it is possible that the presence of vibration, regardless of frequency, affects force control differently for persons with ASD relative to TD controls.

Third, vision of the vibrated effector has been shown to reduce the illusory effect of tendon vibration for gross movements. This phenomenon has not been demonstrated for fine motor behavior, and it is unlikely to affect precision force control because (a) hand and finger posture are consistent during gripping and (b) the hand is in the peripheral visual field during the task. However, to verify that vision of hand posture is not interfering with the effect of tendon vibration, future studies should block participants’ vision of their hand.

The specificity of the interpretation of the effect of tendon vibration on somatosensory feedback processing was limited by (a) the possibility that vibration may have had nonspecific effects on perceived load and muscle stretch in the fingers and (b) the potential influence of cutaneous inputs on the perception of finger position. The present study could not distinguish between the effect of vibration on perceived limb position (muscle stretch) and the perceived force against the fingers (load) as both stimuli are encoded by muscle spindle afferents [56], which are stimulated by the tendon vibrator. Unlike other limbs (e.g., elbow), perception of load in the fingers does not influence position sense [56], and therefore, it is not accurate to assume that vibration induced disruption to load and/or muscle stretch perception is specific to proprioception. Perception of finger posture is also heavily reliant on cutaneous inputs (e.g., skin stretch) [55]. It is possible that TD controls and persons with ASD differentially rely on these other cutaneous cues during fine motor control, which may contribute to between-group differences in motor control across the two vibration conditions. These limitations underscore the need for future research aimed at identifying the contributions of distinct sources of sensory feedback for motor control in persons with ASD. Given findings, including the present findings, suggesting that persons with ASD show reduced integration of non-primary sensory input during motor behavior [13], testing these sensory manipulations across multiple behaviors is necessary to further clarify sensory feedback mechanisms of distinct behavioral impairments in ASD.

## Conclusions

The present study demonstrates that individuals with ASD show a reduced ability to integrate somatosensory feedback during visually guided manual motor behavior implicating deficits integrating multiple sources of sensory feedback to guide precision motor behavior. We also show evidence for atypical development of sensorimotor abilities in ASD characterized by delayed maturation of precision sensorimotor control. These results help clarify the sensory feedback processes contributing to deficits in online motor control in individuals with ASD and provide new insights into important neurodevelopmental processes that contribute to the disorder.

## Data Availability

The datasets used and/or analyzed during the current study are available from the corresponding author on reasonable request.

## Abbreviations

ASD: Autism spectrum disorders
COP: Center of pressure
TD: Typically developing
DSM-V: Diagnostic and Statistical Manual of Mental Disorders, Fifth Edition
ADOS-2: Autism Diagnostic Observation Schedule, Second Edition
ADI-R: Autism Diagnostic Interview – Revised
IQ: Intelligence Quotient
WASI-II: Wechsler Abbreviated Scales of Intelligence; Second Edition
SP (SP-2): Sensory Profile (Second Edition)
BOT-2: Bruininks-Osteretsky Test of Motor Proficiency, Second Edition
MVC: Maximum voluntary contraction
SD: Standard deviation,
SampEn: Sample entropy
MLM: Multilevel linear effects model
PIQ: Perceptual intelligence quotient
VIQ: Verbal intelligence quotient
ADOS-CSS: Autism Diagnostic Observation Schedule – Composite Severity Score
SE: Standard error
FDR: False discovery rate
FC: Functional connectivity
